# From Tissue Architecture To Genetic Signature: Artificial intelligence-based Analysis of Reticulin Framework and Clinical Variables Predicts Molecular Cluster in Paragangliomas

**DOI:** 10.1007/s12022-026-09904-4

**Published:** 2026-02-18

**Authors:** Eleonora Duregon, Mirko Parasiliti-Caprino, Giulia Orlando, Anna Paola Ferrero, Martina Bollati, Rute Pedrosa, Darshan Kumar, Giuseppe Giraudo, Barbara Pasini, Ezio Ghigo, Emanuela Arvat, Marco Volante, Mauro Maccario, Mauro Papotti

**Affiliations:** 1 Department of Oncology, University of Turin, Turin, Orbassano Italy; 2 Arterial Hypertension and Cardiovascular Endocrinology Laboratory, Division of Endocrinology, Diabetes and Metabolism, City of Health and Sciences University Hospital, Turin, Italy; 3 Department of Medical Science, University of Turin, Turin, Italy; 4 Aiforia Technologies Plc, Helsinki, Finland; 5 Division of Surgery, Department of General and Specialized Surgery, City of Health and Sciences University Hospital, Turin, Italy; 6 Division of Medical Genetics, City of Health and Sciences University Hospital, Turin, Italy; 7 Division of Oncological Endocrinology, City of Health and Sciences University Hospital, Turin, Italy

**Keywords:** Paraganglioma, Reticulin framework, Molecular clusters, Artificial intelligence-based morphometric analysis, Computational pathology

## Abstract

Paragangliomas (PGLs), encompassing pheochromocytomas and extra-adrenal paragangliomas, are genetically heterogeneous non-epithelial neuroendocrine neoplasms that segregate into molecular clusters with distinct biological and clinical behavior. Architectural correlates of genotype have not been systematically investigated. This study aimed to assess the reticulin framework as a potential morphologic structural surrogate of molecular background and to introduce a novel deep learning model for its spatially resolved quantitative analysis and genotype prediction. A total of 104 adrenal and extra-adrenal PGLs with complete clinical, pathological, and genetic data were retrospectively analyzed. Reticulin stain was evaluated qualitatively and quantitatively using a supervised convolutional neural network trained on expert-annotated reticulin-stained whole-slide images (WSIs) to map and quantify areas of intact framework and very small nest patterns. Two bias-reduced logistic regression models (Firth’s method) were developed to predict germline cluster 1 genotype, each combining clinical variables (age, tumor size, extra-adrenal presentation) with one artificial intelligence (AI)-derived morphometric feature-percentage of intact framework (Model-INTACT) or very small nests (Model-VSN). PGLs harboring germline cluster 1 variants occurred at a younger age, were larger, more frequently extra-adrenal, and showed significant enrichment of intact reticulin and very small nest patterns compared with cases harboring germline cluster 2 variants and sporadic cases. The supervised AI model accurately mapped and quantified these architectural features across the WSIs. Predictive models integrating AI-derived morphometrics with clinical variables achieved excellent discrimination for germline cluster 1 genotype (AUC 0.981 for Model-INTACT; AUC 0.990 for Model-VSN). Preservation of the reticulin framework, particularly with very small nests, represents a histoarchitectural correlate of pseudohypoxic PGLs. Integration of AI-based morphometric descriptors with clinical parameters enables reliable pre-test prediction of germline cluster 1 genotype, bridging conventional histopathology and molecular classification.

**Supplementary Information:**

The online version contains supplementary material available at 10.1007/s12022-026-09904-4.

## Introduction

Pheochromocytomas and extra-adrenal paragangliomas, collectively known as paragangliomas (PGLs), are rare non-epithelial neuroendocrine neoplasms, with pheochromocytomas arising from the adrenal medulla and extra-adrenal paragangliomas originating from sympathetic or parasympathetic paraganglia located outside the adrenal gland [1–3]. Notably, PGLs exhibit a remarkably high degree of heritability, with approximately 40% to 50% of adult cases and up to 80% of pediatric cases having a hereditary cause, attributable to germline mutations in susceptibility genes, making them one of the most heritable tumor types in humans [1, 4, 5]. This strong genetic basis underscores the need for routine genetic screening on germline DNA of all patients diagnosed with PGLs, regardless of family history, to identify underlying germline susceptibility [6, 7]. However, limitations exist in the ability of current genetic findings to fully predict disease outcomes, coupled with practical challenges such as cost and access worldwide to extensive genetic testing, possibly whole exome [8].

PGLs feature specific molecular subgroups with substantial genotype-phenotype correlations [9–11]. The Cancer Genome Atlas (TCGA) proposed a division into three main molecular expression clusters linked by distinct driver genes [12]. Cluster 1 is associated with the pseudohypoxia pathway and includes two subgroups: tricarboxylic acid cycle (TCA) cycle aberrant (*e*.*g*., *SDHA*, *SDHB*, *SDHC*, *SDHD*, *SDHAF2*, *FH*, *MDH2*, *SUCLG2*) and TCA cycle non-aberrant (e.g., *VHL*, *EPAS1*, *EGLN1*, *EGLN2*) [13]. Cluster 1 typically exhibits a predominant norepinephrine/normetanephrine biochemical profile. However, its secretory phenotype is heterogeneous and may range from non-secretory tumors to dopaminergic, pure noradrenergic, or mixed dopaminergic-noradrenergic profiles. Cluster 2 harbors mutations in kinase-associated pathways (*e*.*g*., *RET*, *NF1*, *TMEM127*, *MAX*, *FGFR1*, *KIF1B*, *HRAS*, *MET*) and is often associated with a higher production of epinephrine/metanephrine and a generally lower metastatic potential compared to cluster 1 [14]. Cluster 3 is characterized by an aberrant Wnt signaling pathway (e.g., somatic mutations in *CSDE1* or *UBTF::MAML3* fusions) and has been shown to exhibit a higher risk of metastatic dissemination [5]. These molecular classifications provide valuable insights into the underlying tumorigenesis, location, and metastatic potential of PGLs [15]. Molecular immunohistochemistry (IHC) plays a critical role in deciphering the genetic basis of PGLs as specific antibodies serve as surrogate markers for underlying genetic alterations [16]. For instance, loss of SDHB immunoreactivity is strongly indicative of germline mutations in *SDHB*, *SDHC*, or *SDHD* genes, while concurrent loss of both SDHA and SDHB suggests an *SDHA* mutation [17–20]. CAIX immunostaining is valuable for identifying *VHL*-mutated PGLs due to its overexpression, and loss or reduced expression of FH, coupled with 2-SC immunoreactivity, points towards *FH* mutations [21, 22]. Alpha-inhibin has been identified as a sensitive marker for cluster 1 PGLs driven by pseudohypoxia-related genes like *SDH*x and *VHL* [23]. In contrast, loss of MAX protein expression can be observed in PGLs with *MAX* mutations [24]. Molecular IHC, in conjunction with morphology and clinical data, is essential for guiding genetic testing, predicting prognosis, and classifying PGLs into molecular subtypes, but some markers, such as alpha-inhibin, are not entirely specific and can be observed in other neoplasms, and other antibodies (for example NF1 and RET) are not sufficiently reliable for mutation detection via IHC [1, 2, 16, 25].

Reticulin stain is a silver-based histochemical stain which renders the fine meshwork of crosslinked connective tissue fibers (mostly type III collagen), known as reticulin framework, which is an essential component of the histological structure of biological tissues. In adrenal gland pathology, reticulin framework assessment is very well characterized with a diagnostic role for neoplastic lesions of the adrenal cortex, including bilateral macronodular adrenocortical disease, adrenocortical adenoma and adrenocortical carcinoma [26]. Accordingly, the reticulin algorithm is a two-step diagnostic method, which distinguishes benign from malignant adrenocortical neoplasms upon the evaluation of reticulin framework alterations paired with the recognition of specific morphological parameters [27, 28]. Unlike lesions of the adrenal cortex, the reticulin framework status of adrenal medulla neoplasms and specifically of PGLs has never been systematically addressed [26]. We, therefore, analyzed the reticulin framework of PGLs and correlated the results with pathological, clinical and genetic data. These observations founded the ground truth for the development of a novel artificial intelligence (AI)-based model to recognize reticulin framework patterns.

## Materials and Methods

### Case Selection

A total of 183 patients with a diagnosis of pheochromocytomas or extra-adrenal paragangliomas between 2000 and 2023 were retrospectively identified from the pathology files of the City of Health and Sciences University Hospital in Turin. Among these, 104 patients (48 males, 46.2%) were eligible for inclusion based on the availability of surgical specimens, complete clinical and biochemical records, and comprehensive germline genetic testing. Patients with partial genetic evaluation (*n* = 61), variants of uncertain significance (*n* = 9), tumors associated with confirmed inherited syndromes not meeting study criteria (*n* = 4), or insufficient clinical information due to death prior to evaluation (*n* = 5) were excluded. Variants of uncertain significance were excluded from the primary analysis to avoid cluster misclassification; their biological interpretation is currently indeterminate and subject to reclassification. Patients dying before surgery could not be included because histopathology and AI morphometrics were unavailable. The final cohort comprised 90 phaeochromocytomas (86.5%) and 14 extra-adrenal paragangliomas (13.5%). Among extra-adrenal tumours, the distribution of sites was: laterocervical (*n* = 10), mediastinal (*n* = 6), interaortico-paraortic abdominal (*n* = 7), and organ of Zuckerkandl (*n* = 4); obviously counts exceed 14 because most patients had multiple lesions/sites.

Before the study started, all cases were de-identified and coded by a pathology staff member not involved in the study, and all data were accessed anonymously. The study was approved by the local Ethical Committee (Protocol No. 0134361) and conducted in accordance with the principles set out in the Declaration of Helsinki.

### Clinical Data

Clinical, biochemical, and imaging data were retrieved from a prospective institutional registry. Parameters collected included age at diagnosis, family history, presenting symptoms, biochemical phenotype, plasma and/or urinary metanephrine levels, chromogranin A (CGA), neuron-specific enolase (NSE), tumor size, localization, presence of bilateral or multifocal disease, and findings from anatomic and functional imaging. Symptom severity was categorized into three groups: asymptomatic, mildly symptomatic (e.g., hypertension, adrenergic symptoms), or severely symptomatic (e.g., hypertensive emergencies, arrhythmias, cerebrovascular events), based on published criteria.

### Genetic Testing

All patients underwent germline genetic testing. From 2005 to 2015, testing was performed using Sanger sequencing and multiplex ligation-dependent probe amplification targeting *RET*, *VHL*, *NF1*, *SDH*x, *TMEM127*, and *MAX*. Since 2015, next-generation sequencing (NGS) panels have been employed, including endocrine tumor susceptibility genes (*RET*, *VHL*, *NF1*, *SDHA*, *SDHAF2*, *SDHB*, *SDHC*, *SDHD*, *TMEM127*, *FH*, *FLCN*, *MAX*, *MDH2*, *MEN1*, *AIP*, *BAP1*, *CDC73*, *DLST*, and *TP53*) and additional cancer predisposition genes. Genetic counseling was provided to all patients prior to testing.

### Histopathologic Evaluation

All hematoxylin and eosin (H&E)–stained slides were reviewed by two pathologists with expertise in endocrine pathology (E.D. and M.P.). Tumors were further classified using the Grading system for Adrenal Pheochromocytoma and Paraganglioma (GAPP). Architectural patterns, vascular and capsular invasion, necrosis, and sustentacular cell distribution were evaluated. Tumor size was based on gross pathology and/or imaging reports.

### Immunohistochemistry

Immunohistochemical stainings were performed on 3-µm sections from representative paraffin blocks using an automated stainer (Ventana BenchMark ULTRA, Roche Diagnostics). Primary antibodies included CGA (clone LK2H10, prediluted, Roche Diagnostics), INSM1 (clone A8, 1:100, Santa Cruz Biotechnology), S100 (polyclonal, prediluted, Roche Diagnostics), SDHB (clone EPR10880, diluted 1:3000, Abcam, Cambridge, UK), and Ki-67 (clone MIB-1, 1:150, Dako-Agilent). SDHB immunostaining was evaluated as positive or negative cytoplasmic granular reactivity and was used as a robust surrogate of *SDHx* loss; routine comprehensive somatic profiling was not performed. The Ki-67 labeling index was evaluated by manual count of 1000 tumor nuclei from printed hotspot of nuclear labeling, and expressed as percentage.

### Reticulin Staining

Reticulin fibers were visualized using the Reticulum II staining kit (Ventana Medical Systems, Roche Diagnostics) on the BenchMark Special Stains platform, following manufacturer protocols. The reticulin framework was assessed at both low (×100) and high (×400) magnification. Within the neoplastic area, reticulin framework was classified as follows: (i) disrupted, when a more or less extensive fragmentation of fibers was observed; (ii) intact, when reticulin framework was made of small to medium-sized nests (range 5–10 cells). A subcategory of intact reticulin framework was featured by a very small nest pattern (range 1–4 cells/nest) [26]. The percentage of tumor area composed of very small nests was also estimated semi-quantitatively. For irregular, partially fused, or tangentially sectioned nests, classification relied primarily on the integrity of the reticulin boundary rather than geometric contour. In borderline areas where cell number or nest outline was ambiguous, the final category was assigned according to the predominant appearance across the involved portion of the nest. All slides were independently reviewed by two expert endocrine pathologists (E.D. and M.P.), and discrepancies were resolved in a consensus session before final classification.

### Imaging and Software

Whole slide images (WSIs) were generated by scanning reticulin-stained slides using a NanoZoomer S210 (Hamamatsu, Shizuoka, Japan) digital slide scanner with ×40 magnification and a resolution of 0.23 μm/pixel. The automatic scanner initialization and the visual inspection after scanning (i.e., verifying focus and absence of artefacts) ensured WSI quality. WSIs (.ndpi extension) were uploaded into Aiforia Create, an image management and analysis cloud platform (Aiforia Technologies, Helsinki, Finland), which supports the development of machine learning models with deep convolutional neural networks.

### AI Model Development and Training

Aiforia Create (Aiforia Technologies, Helsinki, Finland), using a multi-step convolutional neural network (CNN), was employed to perform semantic segmentation of a total of three-layer features. This algorithm firstly segmented tissue from background (CNN 1), after which regions of interest (ROIs) accurately defined morphological areas to identify tumor with an intact reticulin framework (CNN 2), further stratifying it into very small nest pattern (CNN 3). For the algorithm development, 33% (34/104 WSIs) of the total cohort was further divided into training (15/104 WSIs, 14%), testing (17/104 WSIs, 16%) and validation (3/104 WSIs, 3%) datasets. All WSIs used in the training, testing, and validation sets derived from distinct patients, and no WSI was included in more than one dataset. During the training of the models, a total of 2817 accurately selected ROIs were annotated by an expert pathologist over the PGL WSIs and feature labels with the pen tool in Aiforia. The annotated training ROIs were selected to represent intact reticulin framework and very small nest patterns, serving as ground truth for model development. Additional reticulin features, such as fiber thickening or reduplication, were qualitatively reviewed but were not included as separate annotation classes because they were focal and inconsistently represented across cases.

Hyperparameter optimization was performed through both manual and automated procedures. Manual optimization consisted of iterative refinement of annotations and visual inspection of prediction maps, whereas automated adjustments relied on the system’s internal feedback, comparing user annotations with pixel-level verification outcomes. Successive model versions were generated during this iterative process.

The training underwent 5000 iterations, with parameters detailed in Supplementary Tables 1 and Supplementary Table 2. The error rate was calculated as the total number of pixel-level false positives (FP) and false negatives (FN) aggregated across all annotations of the class. Model performance was monitored in an iterative manner, with final error rates below 0.5% for all layers (tissue, intact and very small nest), and a training loss of 0.0029. Performance of the final model was assessed using total area error, precision, sensitivity and F1 score (Supplementary Table 3).

### AI Model Verification and Validation

To evaluate the model performance, the annotations that formed the ground truth were compared with the analytic results of the AI model on 17 reticulin-stained WSIs (275 ROIs) both statistically in Aiforia and visually, all of which were held out from the training set. To validate the AI model, 3 WSIs were used, from which a total of 84 sub-regions were selected (40 ROIs for the intact layer and 44 ROIs for the very small nest layer; Supplementary Table 4). These ROIs were annotated *de novo* by three external pathologists who were not involved in model development, providing the ground truth for the independent validation of the algorithm. Performance metrics were calculated against the manually annotated ground truth from all validators to assess the generalizability of the deep learning pipeline. For the Intact layer, the model achieved an average error in total area of 4.53%, with 98.82% precision, 94.40% sensitivity, and an F1 score of 96.56%. For the Very small nest layer, the model reached an average error in total area of 0.76%, with 100% precision, 97.38% sensitivity, and an F1 score of 98.67%.

### Statistical analysis, Predictive Modelling and Performance Assessment

Distribution of continuous variables was assessed with the Shapiro–Wilk test. Continuous data are reported as median (interquartile range, IQR) and categorical variables as counts (percentages, %). Between-group comparisons used the Mann-Whitney U or Kruskal-Wallis test for continuous variables and the chi-squared or Fisher’s exact test for categorical variables; Dunn’s test with Bonferroni adjustment was applied for multiple comparisons. All tests were two-sided with α = 0.05.

We anticipated small-sample bias and quasi-separation (notably for extra-adrenal paraganglioma and the AI morphometrics); therefore, we fitted Firth’s bias-reduced logistic regression. An initial exploratory backward screen was used only to identify redundancy among candidates; however, the final models were pre-specified to preserve events-per-variable and to reflect clinical/biological plausibility and pre-operative availability: age (years), tumour size (mm), extra-adrenal site (paraganglioma vs. phaeochromocytoma), and one AI morphometric feature (either % intact reticulum area, Model-INTACT, or % very small nests, Model-VSN). Continuous predictors were modelled on their natural scale. Collinearity was assessed via correlation matrices and variance-inflation factors.

Discrimination was quantified using the area under the receiver operating characteristic curve (AUC) derived from model-predicted probabilities. Internal validation used non-parametric bootstrap with 1,000 resamples and refitting within each resample to estimate the sampling distribution of the AUC and obtain 95% confidence intervals (bias-corrected method); bootstrap replicates with non-convergence were excluded from uncertainty estimation. Model calibration was examined graphically using calibration plots (deciles of predicted risk with LOESS smoothing). Decision-analytic performance was explored with decision-curve analysis across clinically plausible risk thresholds. Additional model-comparison metrics (AIC/BIC, Brier score and scaled Brier, calibration intercept/slope, balanced accuracy) were examined and were concordant with the reported results; to prioritise clarity, we present only the performance measures above.

Statistical analyses were performed using Stata version 18.0 (StataCorp, College Station, TX, USA) and GraphPad Prism v9.0 (GraphPad Software, San Diego, CA, USA).

## Results

### Cohort Composition and Molecular Stratification

Within the final cohort of 90 pheochromocytomas and 14 extra-adrenal paragangliomas, a germline mutation was identified in 39 patients (37.5%), including 20 with germline cluster 1 mutations (11 *VHL*, 1 *SDHA*, 8 *SDHB*) and 19 with germline cluster 2 mutations (10 *NF1*, 8 *RET*, 1 *MAX*). No cluster 3 tumors were identified, as somatic mutation analysis was not performed. The remaining 65 cases (62.5%) were classified as sporadic.

### Histopathologic Features and Reticulin Framework Assessment

Tumors measured a median of 42 mm (range, 30–57 mm) and were generally well circumscribed. All cases exhibited conventional morphological features of PGLs, including an organoid architecture composed of large or small nests of tumor cells with peripheral sustentacular cells confirmed by S100 immunostaining. Vascular and/or capsular invasion was observed in 45 tumors (43%), and necrosis was noted in 23 of these. Diffuse expression of CGA and INSM1 was observed in all cases. The median Ki-67 labeling index was 1.8% (range, 0.5–3%).

As previously described, SDHB immunohistochemical staining patterns correlates with the underlying mutational status. In our series of 104 cases, all 10 *SDH*x-mutated tumors lost SDHB expression, whereas all 10 *VHL*-mutated tumors retained SDHB expression. Furthermore, 19 cases harboring *RET*, *NF1*, or *MAX* mutations and 65 sporadic cases were positive for SDHB immunohistochemistry.

**Fig. 1:**
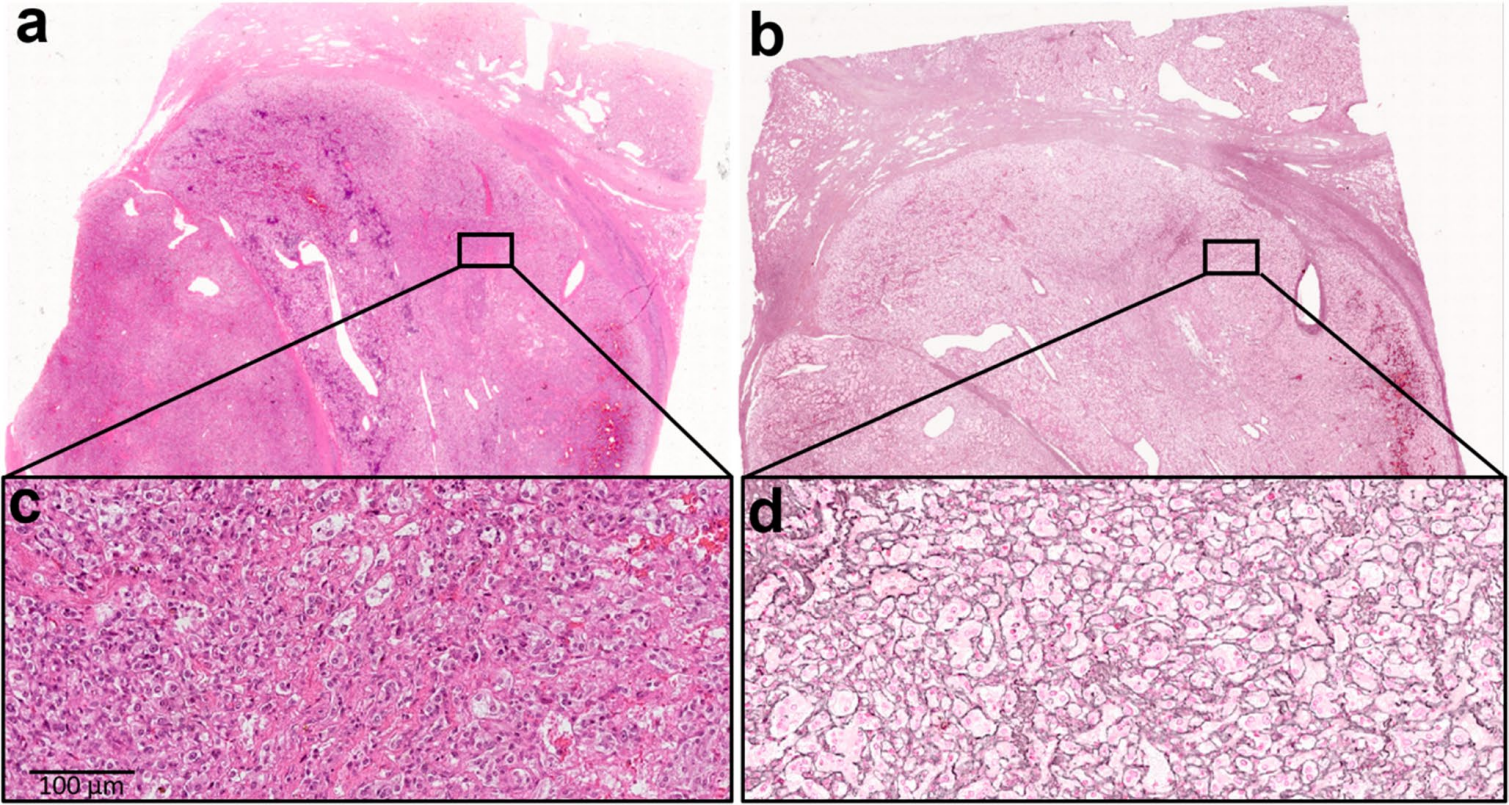
Case of a 24 year-old man, with pheochromocytoma, *VHL* mutation. Reticulin framework is intact with a very small nest pattern (**a**, **c**: hematoxylin and eosin staining; **b**, **d**: reticulin staining)

**Fig. 2:**
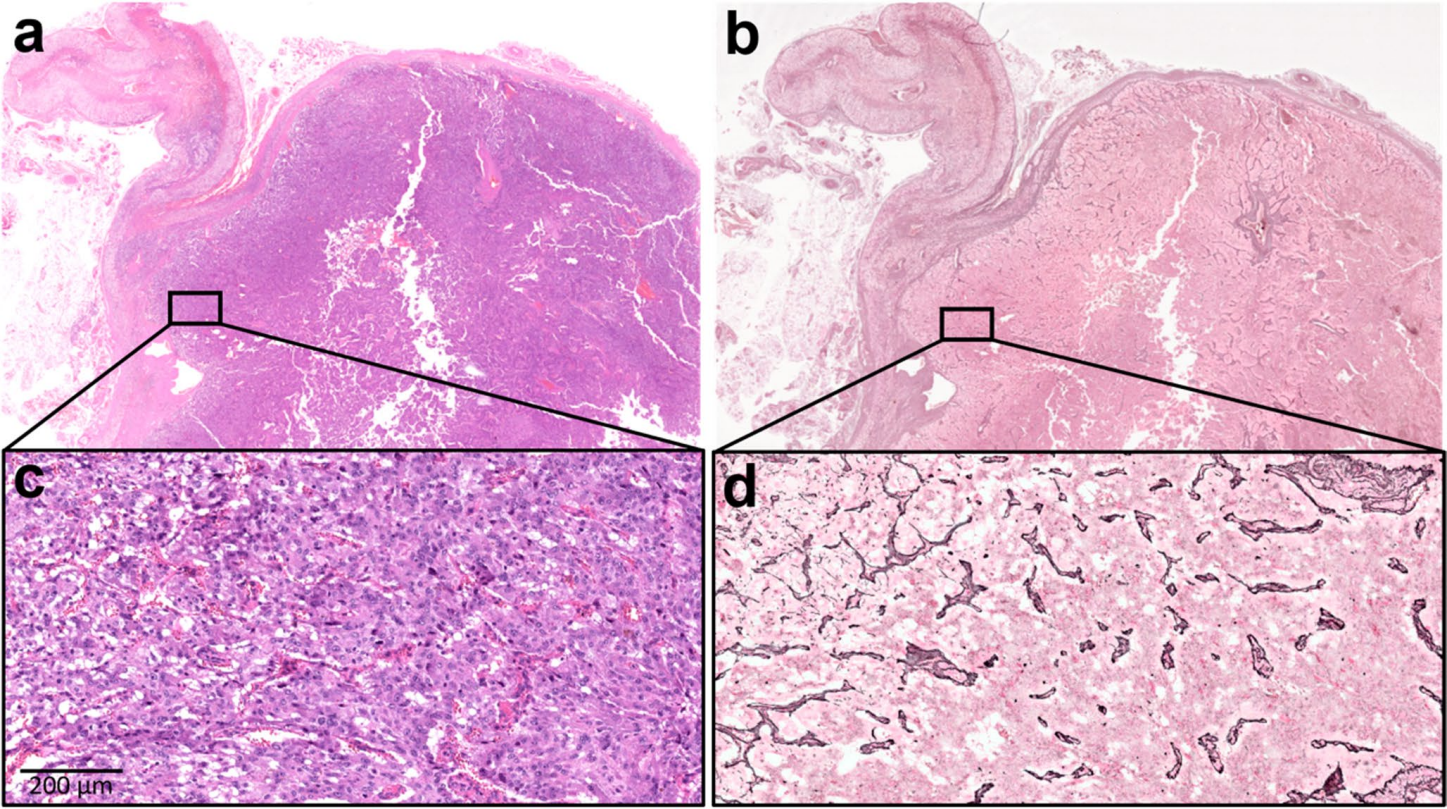
Case of a 52 year-old woman with pheochromocytoma, sporadic. Reticulin framework is extensively disrupted (**a**, **c**: hematoxylin and eosin staining; **b**, **d**: reticulin staining)

Reticulin staining revealed an intact framework in 54 tumors (51.9%), of which 28 exhibited predominantly a very small nest pattern (Fig. 1). Among them, the extent of this pattern involved > 80% of tumor area in 8 cases, 40–79% in 8 cases, and 10–39% in 12 cases. The remaining 50 tumors (48.1%) showed various degrees of reticulin disruption, typically involving most of the tumor area (Fig. 2). Reticulin fibers were generally preserved around medium and large blood vessels. The S100 staining outlined the expected zellballen pattern of sustentacular cells. However, its prominence and distribution varied among cases and did not clearly mirror either the areas of intact reticulin framework or the very small nest pattern.

### Clinico-pathological Correlates of Genetic Background

Patients harboring germline cluster 1 variants were diagnosed at a significantly younger age compared with those with germline cluster 2 variants or sporadic PGLs (median age: 28.0 vs. 45.0 vs. 54.0 years, respectively; *p* < 0.001). This association remained significant when age was analyzed categorically (≤ 40 years; *p* < 0.001). A positive family history of PGLs or related syndromic forms was more frequent among genetically confirmed cases (*p* < 0.001). Plasma and urinary methoxytyramine levels were significantly elevated in germline cluster 1 tumors (*p* = 0.006 and *p* = 0.011, respectively). Regarding the secretory phenotype, patients with germline cluster 1 variants were characterized by a higher prevalence of noradrenergic, dopaminergic, or non-secretory profiles, while patients with germline cluster 2 variants more frequently displayed adrenergic secretion (*p* < 0.001). Tumor size was larger in cases with germline cluster 1 variants, both as a continuous variable (*p* = 0.033) and when using a ≥ 35 mm cut-off (*p* = 0.064). Extra-adrenal paraganglioma presentation was significantly more common in germline cluster 1 variant tumors compared with tumors with germline cluster 2 variants and sporadic cases (*p* < 0.001). Bilateral disease occurred more frequently in patients with tumors harboring germline cluster 1 and cluster 2 variants, with the highest rate in the germline cluster 1 group (*p* = 0.004). Analysis of the reticulin framework revealed that reticulin disruption was significantly more frequent in sporadic PGLs than in tumors with germline cluster 1 or germline cluster 2 variants (*p* < 0.001). The germline cluster 1 variant group showed the lowest rate of disruption (15%) and the highest prevalence and extension of very small nests (*p* = 0.003). Univariate analyses for all clinical and pathological features are shown in Table 1.

**Table 1:** Clinical and pathological features

	Total	Sporadic PGL	Germline cluster 1 variant PGL	Germline cluster 2 variant PGL	p-value^1^
N (%)	104 (100.0%)	65 (62.5%)	20 (19.2%)	19 (18.3%)	
Age at diagnosis (years)	46.5 (32.0-58.0)	54.0 (44.0-64.0)	28.0 (19.5-35.5)	45.0 (29.0-56.0)	<0.001
Male Gender	46.2%	41.5%	60.0%	47.4%	0.348
Family history of PGL	21.2%	3.1%	55.0%	47.4%	<0.001
Signs and Symptoms					0.914
Asymptomatic	28.0%	29.0%	31.6%	21.1%	
Mild	54.0%	51.6%	52.6%	63.2%	
Severe	18.0%	19.4%	15.8%	15.8%	
p-normetanephrine (pmol/L)	3616.0 (2352.6-11888.0)	4471.0 (2352.6-11188.0)	1815.0 (488.2-12888.1)	2592.4 (2480.0-3616.0)	0.893
p-metanephrine (pmol/L)	714.0 (227.0-3814.0)	918.0 (232.0-3666.8)	158.9 (128.1-169.0)	4222.0 (620.4-5698.0)	0.770
p-methoxytyramine (pmol/L)	115.8 (75.0-300.2)	115.8 (75.0-198.6)	10231.3 (76.8-20385.8)	329.7 (75.0-584.4)	0.006
dU-normetanephrine (µg/day)	1812.0 (970.0-3976.0)	2012.5 (986.5-4122.0)	2699.5 (680.0-5297.5)	1443.0 (933.0-2152.0)	0.477
dU-metanephrine (µg/day)	549.0 (168.0-2130.0)	776.0 (216.0-2259.0)	120.0 (88.0-198.0)	730.0 (331.0-1830.0)	0.112
dU-methoxytyramine (µg/day)	291.0 (226.0-605.0)	268.5 (222.5-531.5)	6309.5 (315.0-12304.0)	291.0 (291.0-291.0)	0.011
Secretory phenotype					<0.001
Adrenergic and Noradrenergic	23.0%	28.6%	0.0%	26.7%	
Noradrenergic	42.5%	42.9%	68.8%	13.3%	
Noradrenergic and Adrenergic	24.1%	25.0%	0.0%	46.7%	
Dopaminergic	4.6%	1.8%	12.5%	6.7%	
Non-secretory	5.7%	1.8%	18.8%	6.7%	
CGA (ng/mL)	372.0 (198.0-568.0)	304.0 (131.0-528.0)	449.0 (369.0-600.0)	409.5 (234.5-641.5)	0.474
Positive CGA	85.5%	82.9%	100.0%	80.0%	0.248
NSE (ng/mL)	14.0 (9.9-15.4)	13.6 (9.9-15.7)	14.0 (12.0-14.0)	12.0 (7.2-20.0)	0.839
Positive NSE	18.2%	17.4%	0.0%	33.3%	0.402
Tumor Size (mm)	42.0 (30.0-57.0)	40.0 (30.0-52.5)	51.0 (42.0-67.5)	40.0 (25.0-53.0)	0.033
Adrenal side involvement					0.004
Left	40.2%	46.7%	23.1%	31.6%	
Right	55.4%	53.3%	53.8%	63.2%	
Bilateral	4.3%	0.0%	23.1%	5.3%	
Extra-adrenal paraganglioma presentation at diagnosis	13.5%	9.2%	40.0%	0.0%	<0.001
Ki67%	1.8 (0.5-3.0)	1.5 (0.5-3.0)	1.5 (0.5-3.0)	2.0 (0.5-5.0)	0.107
GAPP Score	3.00 (2.00-5.00)	3.00 (2.00-4.75)	2.00 (1.75-5.00)	3.00 (2.00-4.50)	0.966
Reticulin Framework					<0.001
Present/Intact	51.9%	38.5%	85.0%	63.2%	
Disrupted	48.1%	61.5%	15.0%	36.8%	
Very small nests					0.003
0-9%	73.1%	81.5%	40.0%	78.9%	
10-39%	11.5%	10.8%	15.0%	10.5%	
40-79%	7.7%	3.1%	20.0%	10.5%	
>80%	7.7%	4.6%	25.0%	0.0%	

^1^Significant *p*-value: ≤0.05

Abbreviations: *CGA* chromogranin A, *GAPP* Grading of Adrenal Pheochromocytoma and Paraganglioma, *NSE* neuron specific enolase, *PGL* paraganglioma

### AI-assisted Quantification of Reticulin Patterns

**Fig. 3:**
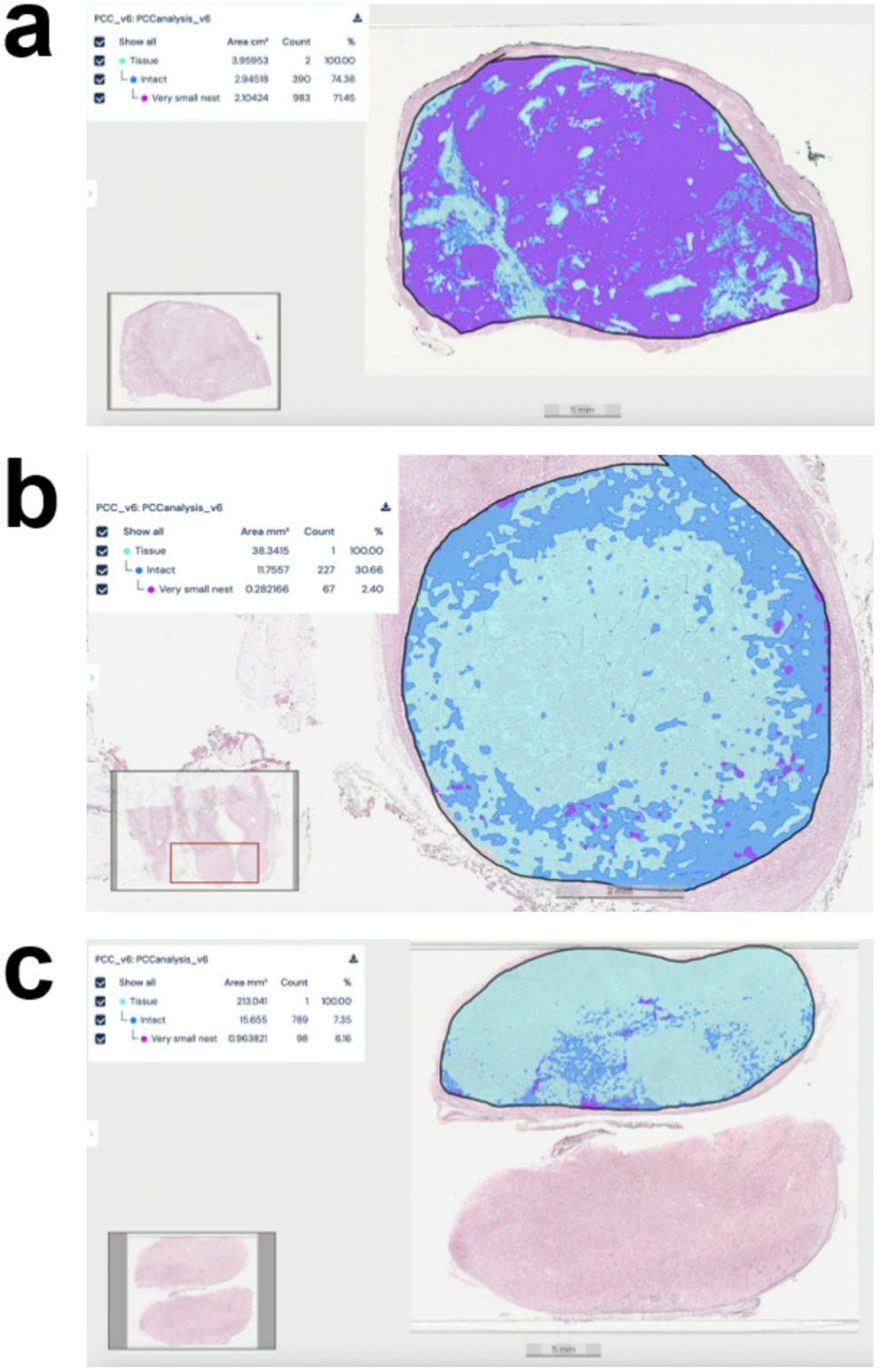
Representative images of three pheochromocytoma cases (**a**, germline cluster 1 variant; **b**, germline cluster 2 variant; **c**, sporadic) analyzed with the Aiforia Create AI model, illustrating the quantification of intact reticulin framework (blue) and very small nests (purple)

**Fig. 4:**
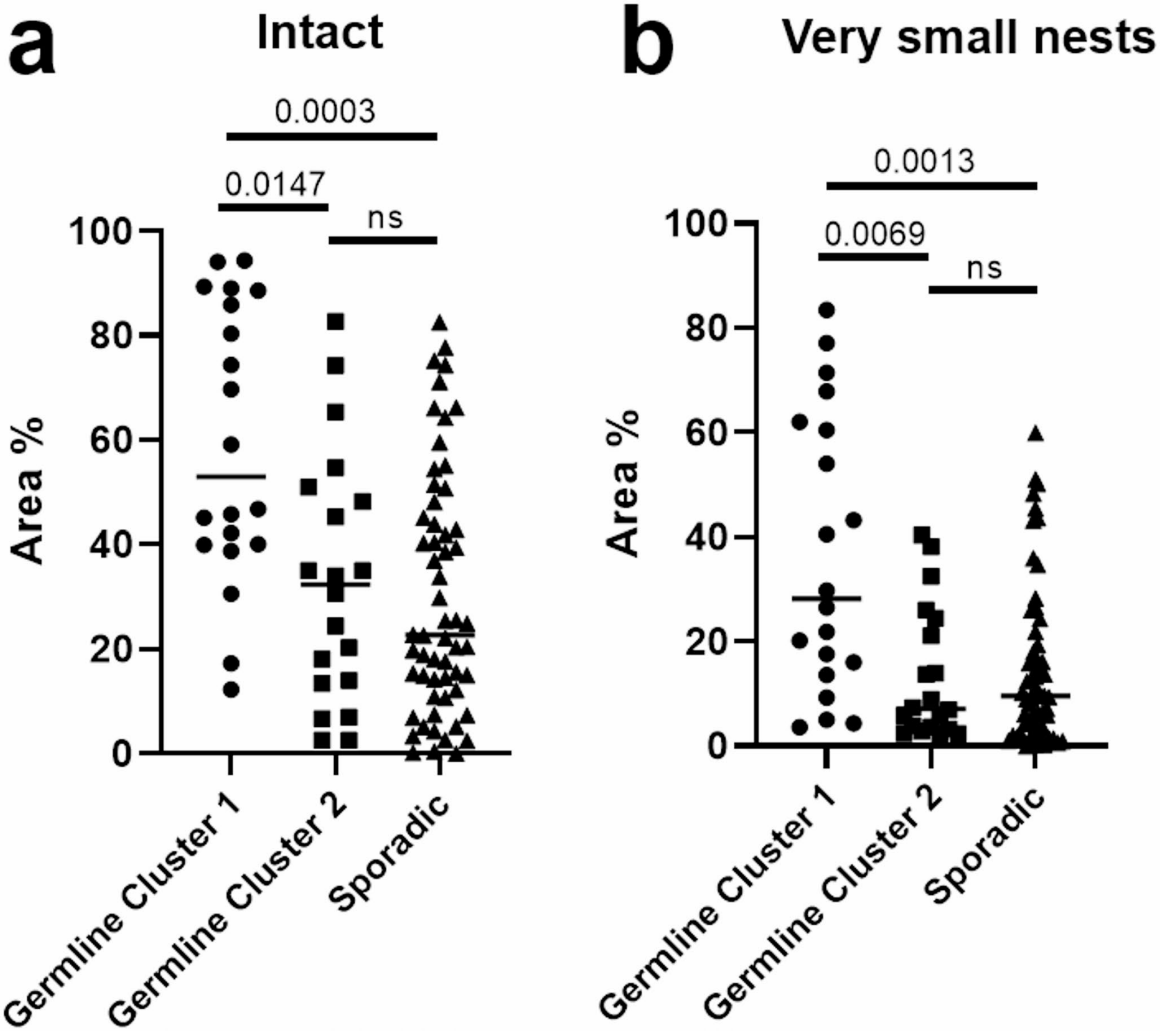
Percentage area comparison of intact reticulin (**a**) and very small nest patterns (**b**) among PGL harboring germline cluster 1 variants, germline cluster 2 variants and sporadic PGLs

The AI model was applied to the entire study cohort. Quantitative output included the percentage area of intact reticulin and very small nest patterns for each WSI (Fig. 3). When analyzed as a continuous variable, the median percentage of tumor area composed of very small nests was 35.2% (range 16.0–62.0) in germline cluster 1, compared with 7.3% (range 3.7–24.4) in germline cluster 2 and 9.4% (range 3.1–21.9) in sporadic tumors. Similarly, the median percentage of intact reticulin framework was 53.0% (range 40.1–88.7) in germline cluster 1 versus 30.7% (range 13.5–48.2) in germline cluster 2 and 20.5% (range 7.4–40.4) in sporadic tumors. Following stratification by molecular cluster, a significantly higher amount of intact reticulin framework was observed in germline cluster 1 compared with germline cluster 2 (*p* = 0.015) and sporadic tumors (*p* = 0.0003), with no significant difference between germline cluster 2 and sporadic tumors (Fig. 4a). A similar distribution was noted for the very small nest pattern, which was significantly more prevalent in germline cluster 1 compared with germline cluster 2 (*p* = 0.007) and sporadic tumors (*p* = 0.001). No significant difference was found between germline cluster 2 and sporadic tumors (Fig. 4b).

### Predictive Modeling of Germline Cluster 1 Genotype Using AI-derived Morphometrics

Two bias-reduced logistic regression models were developed to predict the presence of a germline cluster 1 genotype by integrating clinical variables with AI-derived morphometric features. Both models demonstrated excellent discriminatory performance after internal bootstrap validation. The Model-INTACT, incorporating the percentage area of intact reticulin framework, achieved an AUC of 0.981 (bootstrap 95% CI, 0.940–1.000; bias-corrected; 1,000 replicates). The bootstrap standard error was 0.012, and the estimated bias was + 0.0025 (Supplementary Table 5). The Model-VSN, incorporating the percentage area of very small nests within the intact reticulin regions, achieved an AUC of 0.990 (bootstrap 95% CI, 0.962–1.000; bias-corrected; 993 valid replicates, 7 non-convergent). The bootstrap standard error was 0.007, and the estimated bias was + 0.0018 (Supplementary Table 6). In both models, younger age, larger tumor size, extra-adrenal paraganglioma presentation, and higher AI-derived area metrics were directionally associated with an increased probability of harboring a germline cluster 1 genotype. Models are provided in the Supplementary Materials (Supplementary Figs. 1 and 2).

## Discussion

In this study, we found that the reticulin framework in PGLs displays reproducible architectural features reflecting their molecular background. We identified intact reticulin framework and areas composed of very small nests as the two main architectural patterns, both strongly associated with germline cluster 1 (pseudohypoxic) variants. Conversely, tumors harboring germline cluster 2 variants and apparently sporadic neoplasms more often showed a disrupted framework.

PGLs stratification in clusters has direct clinical relevance, as tumors harboring germline cluster 1 variants typically occur at a younger age, are more often multifocal, and carry a higher risk of metastatic behavior [3], making genotype-based classification integral to prognostic assessment and management. In routine practice, IHC can act as a surrogate when comprehensive genetic testing is unavailable. In particular, SDHB IHC is pivotal to infer SDHx dysfunction: loss of SDHB expression correlates strongly with inactivating *SDH*x mutations and supports allocation to the pseudohypoxia cluster [29]. Our series reproduced this association with complete concordance between SDHB loss and *SDH*x mutation, and retained SDHB expression in *VHL*-mutated and sporadic cases, aligning with prior multicenter data [19, 20, 29].

While IHC captures targeted molecular surrogates, it does not encompass tissue architecture and stromal context. Accordingly, there is growing interest in the tumor microenvironment (TME) as an active driver of PGL biology, especially in pseudohypoxic tumors in which normoxic HIF-2α stabilization reshapes metabolic and immune programs. Pseudohypoxic PGLs display an immunosuppressive/“cold” phenotype, with reduced CD8 + infiltration and lower PD-L1 expression, as shown by IHC and transcriptomics [30]. Mechanistically, HIF-2α-driven metabolic rewiring (e.g., LDHA up-regulation) and catecholamine pathway shifts (e.g., PNMT down-regulation) are implicated in this immune exclusion [30].

In this context, the extracellular matrix, and specifically reticulin fibers, represents a histologic readout of tumor–stroma interactions. Reticulin staining is well established in adrenal cortical pathology, as part of diagnostic algorithms and carcinoma delineation, and has targeted applications in other endocrine organs [26–28]. However, a systematic appraisal in PGLs has been lacking. We addressed this gap by integrating qualitative assessment with AI-assisted quantification of reticulin framework integrity and very small nests.

Our findings show that tumors harboring germline cluster 1 variants more frequently preserve an intact reticulin framework and enrich for very small nests, whereas sporadic tumors more commonly exhibit reticulin disruption. Although not pathognomonic and with partial overlap between molecular groups at intermediate values, these architectural correlates showed a strong genotype-dependent distribution and paralleled the clinical and biochemical phenotype characteristic of germline cluster 1 disease (younger age at diagnosis, higher frequency of extra-adrenal paraganglioma, noradrenergic or silent secretion). The concordance between morphology (reticulin patterns), IHC (SDHB), and genotype underscores the potential of histologic surrogates to inform risk stratification in diagnostic settings, particularly where molecular testing is limited [29].

From a biological standpoint, the enrichment of intact reticulin framework and very small nests in germline cluster 1 variant PGLs likely reflects broader tumor-stroma interactions driven by pseudohypoxic signalling rather than a single pathway effect. Current PGL models, including rodent-derived cell lines, xenograft models, and patient-derived primary cultures, recapitulate key metabolic, mitochondrial, and HIF-2α–dependent pseudohypoxic programs, but none provide quantitative or structural characterization of the reticulin scaffold [31, 32]. Recent studies on the PGL tumor microenvironment further show that pseudohypoxic tumors exhibit pronounced extracellular matrix remodeling, altered fibrillar collagen composition, stromal activation, and distinctive angiogenic programs, particularly in *SDHx*-mutated lesions [33, 34]. These processes are biologically compatible with the preservation of a fine, continuous reticulin scaffold supporting small, tightly organized nests, although no experimental system has yet linked pseudohypoxic signaling to this specific architectural phenotype.

Accordingly, our observations should be interpreted as morphologic correlates of pseudohypoxic biology rather than mechanistic evidence, and may also reflect the contribution of broader tumor-stroma interactions, including stromal remodeling, tumor growth kinetics, and vascular scaffolding effects. These findings highlight the need for future PGL models that preserve cytoarchitecture and matrix organization to investigate stromal-tumor interactions at this resolution.

In parallel with our findings, a growing body of literature supports the use of artificial intelligence within computational pathology to extract clinically relevant architectural information from routine histopathology in endocrine and neuroendocrine tumors. Recent studies in pancreatic neuroendocrine tumors have shown that quantitative analysis of tumor architecture using deep learning provides prognostic stratification beyond conventional grading, underscoring the value of objective architectural assessment [35]. Similarly, work in papillary thyroid carcinoma has demonstrated that analysis of routine hematoxylin–eosin sections can identify morphologic patterns associated with recurrent driver genetic alterations, including *BRAF*- and *RAS*-related genotypes [36]. Together, these studies support the concept that information related to tumor genotype is, at least in part, reflected in tissue architecture and can be captured through computational analysis.

Within this context, our study is aligned with these approaches but differs in both biological focus and methodological emphasis. Rather than examining epithelial tumor features or tumor-parenchymal interfaces on H&E sections, we focused on the reticulin framework as a stromal and architectural scaffold, using a dedicated histochemical stain combined with quantitative image analysis. By relating measures of reticulin organization to molecular clusters, we extend existing work by showing that stromal architecture may also convey information related to underlying pseudohypoxic programs in PGLs. Taken together, these findings raise the possibility that extracellular matrix organization contributes to the morphologic features associated with specific molecular backgrounds in endocrine tumors.

Because architectural appraisal may be affected by interobserver variability or suboptimal stain quality, we implemented a CNN-based pipeline to standardize quantification. The model segmented tissue, identified areas with intact reticulin and then the subset showing very small nests, excluding non-informative regions (hemorrhage, vessels and fibrotic stroma). Quantitative outputs recapitulated manual assessment, with higher proportions of intact framework and very small nests in germline cluster 1 variant tumors than in germline cluster 2 variant or sporadic tumors. Building on these quantitative results, we combined the AI-derived morphometric descriptors with simple clinical variables - age, tumor size, and extra-adrenal paraganglioma presentation - to develop predictive models for germline cluster 1 variant status. Both models achieved excellent discriminatory performance, with AUCs of 0.981 for the intact reticulin framework and 0.990 for the very small nest descriptor. The slightly superior performance of the latter suggests that the abundance of very small nests may capture a fine-grained architectural signal particularly enriched in germline cluster 1 (pseudohypoxic) tumors. The directions of effect for the clinical covariates - younger age, larger lesion size, and extra-adrenal paraganglioma presentation - were consistent with the known phenotype of germline cluster 1 variant PGLs, supporting the biological plausibility of the models. Methodologically, bias-reduced logistic regression and bootstrap validation ensured stability and minimal bias despite the modest sample size.

Although the segmentation model achieved very high internal performance metrics, these results should be interpreted in the context of a limited independent validation set. Accordingly, the CNN is best regarded as a single-center, proof-of-concept tool developed to support quantitative exploration of reticulin architecture rather than immediate clinical deployment. Broader generalizability will require future multi-center, multi-scanner validation on larger and more heterogeneous datasets.

These findings indicate that adding a simple, quantitative AI-derived feature to routine clinical variables can substantially enhance genotype prediction in PGLs, providing an accessible and reproducible framework for pre-test risk stratification. Among the two descriptors, very small nests showed slightly superior performance and may be preferred when both metrics are available. Future studies should pursue external validation across centers and scanners, and assess the clinical utility of these models within real-world genetic testing workflows.

From a clinical standpoint, our data suggest two practical implications. First, routine reticulin staining can provide additional morphological context when evaluating PGLs with suspected pseudohypoxic background, potentially prioritizing cases for genetic counseling/testing. Second, AI-assisted morphometrics offers a scalable, observer-independent tool to standardize this readout and integrate it into digital workflows, which may be particularly valuable in resource-constrained settings.

This study has limitations. It is a single-center, retrospective analysis, and somatic profiling was not performed, precluding identification of cluster 3 tumors. In addition, sporadic PGLs were not further stratified according to molecular cluster. Therefore, the present analysis specifically informs the clinicopathological correlates of the reticulin framework in tumors harboring germline cluster 1 and germline cluster 2 variants. External validation of the AI-based segmentation model was performed on a limited number of cases. Broader validation across centers, scanners, and staining protocols will be required to formally assess robustness and generalizability. Finally, architectural correlates should be interpreted as probabilistic morphologic surrogates rather than deterministic biomarkers.

In summary, we describe a morphology–genotype correlation in PGLs whereby preservation of the reticulin framework, particularly with very small nests, is enriched in tumors harboring germline cluster 1 variants. An AI-assisted approach reliably quantifies these features, mirrors expert assessment, and achieves high predictive performance for germline cluster 1 status when combined with clinical variables. These insights advocate for bridging conventional histopathology with computational analysis to surface latent diagnostic signals in routine stains, refine pre-test probability for genetics, and ultimately support personalized care pathways in PGLs [3, 5, 26, 29, 30, 37–42].

## Supplementary Information

Below is the link to the electronic supplementary material.

Supplementary Material 1

Supplementary Material 2

## Data Availability

No datasets were generated or analysed during the current study.
