# Supplementary material for: From Tissue Architecture To Genetic Signature: Artificial intelligence-based Analysis of Reticulin Framework and Clinical Variables Predicts Molecular Cluster in Paragangliomas
Source: Endocr Pathol. 2026 Feb 18;37(1):8. doi: 10.1007/s12022-026-09904-4 (PMC12916514; doi:10.1007/s12022-026-09904-4)

**From tissue architecture to genetic signature: artificial intelligence-based analysis of reticulin framework and clinical variables predicts molecular cluster in paragangliomas**

Eleonora Duregon^1*^, Mirko Parasiliti-Caprino^2,3*^, Giulia Orlando^1^, Anna Paola Ferrero^3^, Martina Bollati^2^, Rute Pedrosa^4^, Darshan Kumar^4^, Giuseppe Giraudo^5^, Barbara Pasini^6^, Ezio Ghigo^2,3^, Emanuela Arvat^3,7^, Marco Volante^1^, Mauro Maccario^2,3^ and Mauro Papotti^1^

^*^Eleonora Duregon and Mirko Parasiliti-Caprino contributed equally to this work.

^1^Department of Oncology, University of Turin, Orbassano, Turin, Italy.

^2^Arterial Hypertension and Cardiovascular Endocrinology Laboratory, Division of Endocrinology, Diabetes and Metabolism, City of Health and Sciences University Hospital, Turin, Italy.

^3^Department of Medical Science, University of Turin, Turin, Italy.

^4^Aiforia Technologies Plc, Helsinki, Finland.

^5^Division of Surgery, Department of General and Specialized Surgery, City of Health and Sciences University Hospital, Turin, Italy.

^6^Division of Medical Genetics, City of Health and Sciences University Hospital, Turin, Italy.

^7^Division of Oncological Endocrinology, City of Health and Sciences University Hospital, Turin, Italy.

**Corresponding author:**

Dr. Eleonora Duregon, M.D., Ph.D.

Pathology Unit, City of Health and Sciences University Hospital, Turin, Italy

Department of Oncology, University of Turin

Regione Gonzole, 10 - 10043 Orbassano (TO), Italy

Email: eleonora.duregon@unito.it

**Supplementary Figures**

**Supplementary Fig. 1.** Receiver-operating characteristic (ROC) for Model-INTACT. ROC curve of the Firth bias-reduced logistic model predicting cluster 1 genotype from age, tumor size, extra-adrenal paraganglioma at diagnosis (vs adrenal pheochromocytomas), and the AI-derived % intact reticulum area; axes show sensitivity versus 1–specificity. The model shows excellent discrimination (AUC = 0.981; bootstrap 95% CI, 0.940–1.000; bias-corrected; 1,000 replicates). *Abbreviations*: AUC, area under the curve.


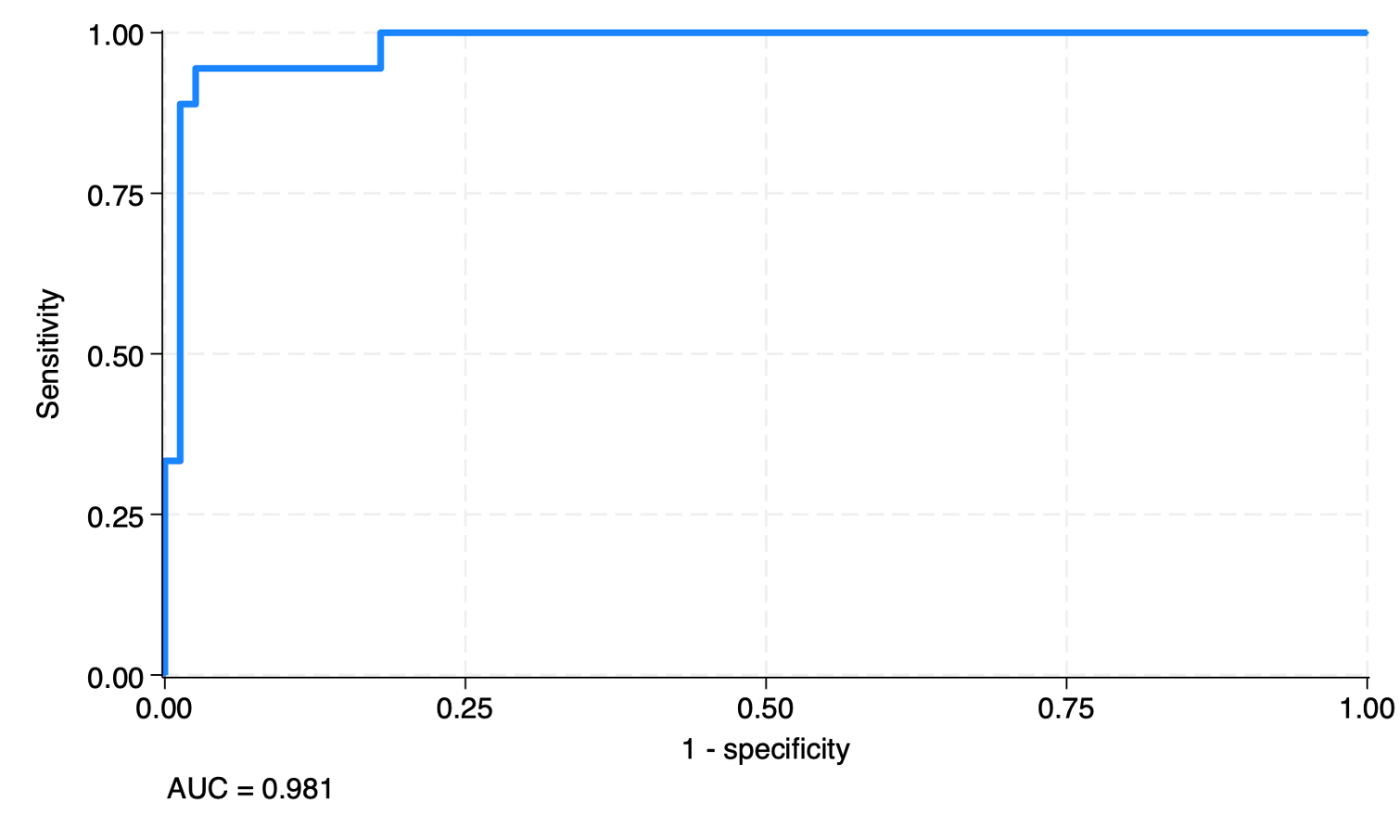


**Supplementary Fig. 2.** Receiver-operating characteristic (ROC) for Model-VSN. ROC curve of the Firth bias-reduced logistic model predicting cluster 1 genotype from age, tumor size, extra-adrenal paraganglioma at diagnosis (vs adrenal pheochromocytomas), and the AI-derived % very small nests; axes show sensitivity versus 1–specificity. The model shows near-perfect discrimination (AUC = 0.990; bootstrap 95% CI, 0.962–1.000; bias-corrected; 993 valid replicates, 7 non-convergent). *Abbreviations*: AUC, area under the curve.
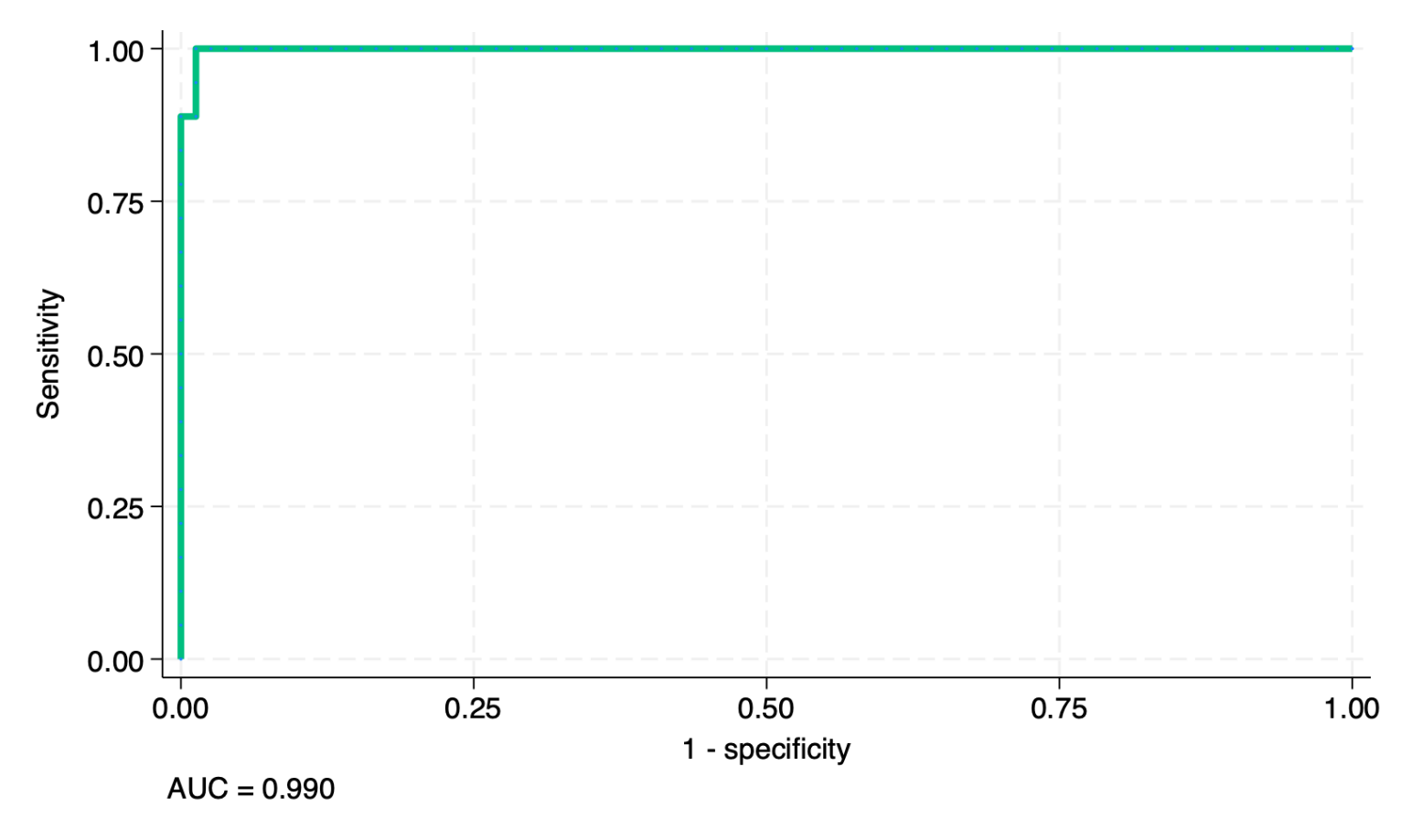

Supplement: Supplementary file 1 — Supplementary Material 1 [file 12022_2026_9904_MOESM1_ESM.docx]
