# Supplementary material for: From Tissue Architecture To Genetic Signature: Artificial intelligence-based Analysis of Reticulin Framework and Clinical Variables Predicts Molecular Cluster in Paragangliomas
Source: Endocr Pathol. 2026 Feb 18;37(1):8. doi: 10.1007/s12022-026-09904-4 (PMC12916514; doi:10.1007/s12022-026-09904-4)
Supplement: Supplementary file 2 — Supplementary Material 2 [file 12022_2026_9904_MOESM2_ESM.docx]

**From tissue architecture to genetic signature: artificial intelligence-based analysis of reticulin framework and clinical variables predicts molecular cluster in paragangliomas**

Eleonora Duregon^1*^, Mirko Parasiliti-Caprino^2,3*^, Giulia Orlando^1^, Anna Paola Ferrero^3^, Martina Bollati^2^, Rute Pedrosa^4^, Darshan Kumar^4^, Giuseppe Giraudo^5^, Barbara Pasini^6^, Ezio Ghigo^2,3^, Emanuela Arvat^3,7^, Marco Volante^1^, Mauro Maccario^2,3^ and Mauro Papotti^1^

^*^Eleonora Duregon and Mirko Parasiliti-Caprino contributed equally to this work.

^1^Department of Oncology, University of Turin, Orbassano, Turin, Italy.

^2^Arterial Hypertension and Cardiovascular Endocrinology Laboratory, Division of Endocrinology, Diabetes and Metabolism, City of Health and Sciences University Hospital, Turin, Italy.

^3^Department of Medical Science, University of Turin, Turin, Italy.

^4^Aiforia Technologies Plc, Helsinki, Finland.

^5^Division of Surgery, Department of General and Specialized Surgery, City of Health and Sciences University Hospital, Turin, Italy.

^6^Division of Medical Genetics, City of Health and Sciences University Hospital, Turin, Italy.

^7^Division of Oncological Endocrinology, City of Health and Sciences University Hospital, Turin, Italy.

**Corresponding author:**

Dr. Eleonora Duregon, M.D., Ph.D.

Pathology Unit, City of Health and Sciences University Hospital, Turin, Italy

Department of Oncology, University of Turin

Regione Gonzole, 10 - 10043 Orbassano (TO), Italy

Email: eleonora.duregon@unito.it

**Supplementary Methods**

**AI Model Architecture and Training Details**

The AI model was developed using the Aiforia Create platform (Aiforia Technologies, Helsinki, Finland) and implemented as a multi-step semantic segmentation pipeline optimized for histopathology image analysis. The workflow consisted of three hierarchical convolutional neural network layers trained sequentially: tissue detection (CNN1), intact reticulin framework segmentation (CNN2), and very small nest pattern segmentation (CNN3).

The underlying architecture is based on a proprietary encoder-decoder design tailored for region-based semantic segmentation. All models were trained from scratch within the Aiforia environment and were not initialized from external pre-trained weights. Training was performed on manually annotated regions of interest, as detailed in Supplementary Table 1.

Training and inference were conducted at fixed magnification levels defined by the platform. Image tiling, training window size, and overlap were handled internally by the software and were not manually specified. The effective field of view and training window size ranges used by the platform are reported in Supplementary Table 2.

Data augmentation strategies applied during training included rotation, flipping, scaling, contrast adjustment, luminance variation, and blur, as summarized in Supplementary Table 2. The “complexity” parameter reflects the combined effect of network depth and number of neurons per layer, rather than the number of layers alone.

Annotation rules for intact reticulin framework and very small nests followed the criteria described in the main Methods section. In brief, classification was based on the integrity of the reticulin boundary, with ambiguous or irregular areas assigned according to the predominant pattern within the region.

Prediction outputs were generated as probability maps and post-processed to calculate area-based quantitative metrics. Non-informative regions, including hemorrhage, large vessels, and fibrotic stroma, were excluded from analysis prior to area computation.

As several components of the pipeline, including the exact network architecture, optimization routines, and image tiling strategy, are proprietary to the Aiforia Create platform, the model cannot be independently reimplemented outside this environment. Reproducibility is therefore ensured at the level of annotation strategy, workflow design, and quantitative output generation.

**Supplementary Tables**

**Supplementary Table 1.** Training ground truth.

| **CNN** | **Total number of ROIs** | **Total Area of the annotated region** | **Total number of images used** |
| --- | --- | --- | --- |
| Tissue (CNN1) | 2817 | 657.859 mm^2^ | 15 |
| Intact (CNN2) |  | 30.447 mm^2^ | 15 |
| Very small nests (CNN3) |  | 8.815 mm^2^ | 11 |

*Abbreviations*: CNN, convolutional neural network; ROIs, regions of interest.

**Supplementary Table 2.** CNN details (Aiforia model hyperparameters).

|  | | **Layers (CNNs)** | | |
| --- | --- | --- | --- | --- |
|  |  | **Tissue (CNN1)** | **Intact (CNN2)** | **Very small nests (CNN3)** |
| **Type (semantic segmentation)** | | Region | Region | Region |
| **Complexity*** | | Complex | Extra Complex | Extra Complex |
| **Field of View** | | 200 $\mu m$ | 80 $\mu m$ | 80 $\mu m$ |
| **Training parameters** | **Weight decay** | 0.0001 | 0.0001 | 0.0001 |
|  | **Mini-batch size** | Default | 20 | 20 |
|  | **Mini-batches per iteration** | 20 | 20 | 20 |
|  | **Iterations Without progress** | Default | 1000 | Default |
|  | **Initial learning rate** | 1 | 1 | 1 |
| **Image augmentation** | **Scale (min/max)** | -1/1.01 | -1/1.01 | -1/1.01 |
|  | **Aspect ratio** | 1 | 1 | 1 |
|  | **Maximum shear** | 1 | 1 | 1 |
|  | **Luminance (min/max)** | -1/1 | -1/1 | -1/1 |
|  | **Contrast (min/max)** | -1/1.01 | -1/1.01 | -1/1.01 |
|  | **Max with balance change** | 1 | 1 | 1 |
|  | **Noise** | 0 | 0 | 0 |

*Abbreviations*: CNN, convolutional neural network.

* The “complexity” parameter is directly proportional to the number of layers and the number of neurons per layer in the CNN architecture.

**Supplementary Table 3.** Performance Metrics of the convolutional neural network (CNN).

| **Metric** | **Tissue** | **Intact** | **Very small nests** |
| --- | --- | --- | --- |
| Precision, % | 99.98 | 99.34 | 98.63 |
| Sensitivity, % | 99.89 | 99.78 | 98.83 |
| F1 score, % | 99.94 | 99.56 | 98.73 |
| Total area error, % | 0.10 | 0.30 | 0.40 |
| Error (false positive/false negative), % | 0.13 (0.02/0.11) | 0.88 (0.66/0.22) | 2.55 (1.37/1.17) |
| False positive, % | 0.02 | 0.66 | 1.37 |
| False negative, % | 0.11 | 0.22 | 1.17 |

**Supplementary Table 4.** Validation ground truth.

| **CNN** | **Total number of ROIs** | **Total Area of the annotated region** | **Total number of images used** |
| --- | --- | --- | --- |
| Intact (CNN2) | 84 (25 intact, 30 very small nest and 29 background areas) | 0.787 mm^2^ | 3 |
| Very small nests (CNN3) |  | 0.454 mm^2^ | 3 |

*Abbreviations*: CNN, convolutional neural network; ROIs, regions of interest.

**Supplementary Table 5**. Predictive model for PGL harboring germline cluster 1 variants, Model-INTACT (AI: % intact reticulum area).

Firth’s bias-reduced logistic regression including age at diagnosis (years), tumor size (mm), extra-adrenal paraganglioma at diagnosis (pheochromocytomas vs extra-adrenal paragangliomas), and the AI-derived percentage of intact reticulum area. Coefficients are penalized log-odds; exponentiating yields odds ratios. Continuous effects are per 1 year, 1 mm, and 1 percentage point increase, respectively. Two-sided p values and 95% CIs are from the penalized likelihood. Internal discrimination: AUC 0.981 (bootstrap bias-corrected 95% CI 0.940–1.000, 1,000 resamples).

| PGL harboring germline cluster 1 variants | β-coeff | 95% CI of β | OR | 95% CI of OR | p-value |
| --- | --- | --- | --- | --- | --- |
| Age (years) | -0.159 | -0.245 to -0.073 | 0.853 | 0.783–0.930 | <0.001 |
| Tumor size (mm) | 0.037 | -0.002 to 0.075 | 1.038 | 0.998–1.078 | 0.064 |
| Extra-adrenal paraganglioma presentation at diagnosis | 2.856 | 0.317 to 5.394 | 17.392 | 1.373–220.082 | 0.028 |
| AI - % of intact area | 0.069 | 0.024 to 0.115 | 1.071 | 1.024–1.122 | 0.003 |

*Abbreviations*: CI, confidence interval; AUC, area under the ROC curve; coeff: coefficient, OR: odds ratio.

**Supplementary Table 6**. Predictive model for PGL harboring germline cluster 1 variants, Model-VSN (AI: % very small nests).

Firth’s bias-reduced logistic regression including age at diagnosis (years), tumor size (mm), extra-adrenal paraganglioma at diagnosis (pheochromocytomas vs extra-adrenal paragangliomas), and the AI-derived percentage of very small nests. Coefficients are penalized log-odds; exponentiating yields odds ratios. Continuous effects are per 1 year, 1 mm, and 1 percentage point increase, respectively. Two-sided p values and 95% CIs are from the penalized likelihood. Internal discrimination: AUC 0.990 (bootstrap bias-corrected 95% CI 0.962–1.000, 993 valid resamples).

| PGL harboring germline cluster 1 variants | β-coeff | 95% CI of β | OR | 95% CI of OR | p-value |
| --- | --- | --- | --- | --- | --- |
| Age (years) | -0.296 | -0.597 to 0.006 | 0.744 | 0.550–1.006 | 0.054 |
| Tumor Size (mm) | 0.044 | 0.007 to 0.082 | 1.045 | 1.007–1.085 | 0.022 |
| Extra-adrenal paraganglioma presentation at diagnosis | 5.480 | 0.853 to 10.107 | **239.847** | 2.347–24514.008 | 0.020 |
| AI - % of Very Small Nests | 0.139 | 0.015 to 0.264 | 1.149 | 1.015–1.302 | 0.029 |

*Abbreviations*: CI, confidence interval; AUC, area under the ROC curve, coeff: coefficient, OR: odds ratio.
